# Synergy of Dietary Quercetin and Vitamin E Improves Cecal Microbiota and Its Metabolite Profile in Aged Breeder Hens

**DOI:** 10.3389/fmicb.2022.851459

**Published:** 2022-05-17

**Authors:** Felix Kwame Amevor, Zhifu Cui, Xiaxia Du, Jing Feng, Gang Shu, Zifan Ning, Dan Xu, Xun Deng, Weizhen Song, Youhao Wu, Xueqing Cao, Shuo Wei, Juan He, Fanli Kong, Xiaohui Du, Yaofu Tian, Benjamin Karikari, Diyan Li, Yan Wang, Yao Zhang, Qing Zhu, Xiaoling Zhao

**Affiliations:** ^1^Farm Animal Genetic Resources Exploration and Innovation Key Laboratory of Sichuan Province, Sichuan Agricultural University, Chengdu, China; ^2^Institute of Animal Husbandry and Veterinary Medicine, College of Agriculture and Animal Husbandry, Tibet Autonomous Region, China; ^3^Department of Basic Veterinary Medicine, Sichuan Agricultural University, Chengdu, China; ^4^College of Life Science, Sichuan Agricultural University, Ya’an, China; ^5^Key Laboratory of Biology and Genetics and Breeding for Soybean, Nanjing Agricultural University, Nanjing, China

**Keywords:** aged breeder hens, gut microbiota, metabolite, dietary quercetin, vitamin E

## Abstract

In the present study, the synergistic effects of quercetin (Q) and vitamin E (E) on cecal microbiota composition and function, as well as the microbial metabolic profile in aged breeder hens were investigated. A total of 400 (65 weeks old) Tianfu breeder hens were randomly allotted to four experimental groups (four replicates per group). The birds were fed diets containing quercetin at 0.4 g/kg, vitamin E (0.2 g/kg), quercetin and vitamin E (QE; 0.4 g/kg and 0.2 g/kg), and a basal diet for a period of 10 wks. After the 10 week experimental period, the cecal contents of 8 aged breeder hens per group were sampled aseptically and subjected to high-throughput 16S rRNA gene sequencing and untargeted metabolomic analysis. The results showed that the relative abundances of phyla *Bacteroidota*, *Firmicutes,* and *Actinobacteriota* were the most prominent among all the dietary groups. Compared to the control group, the relative abundance of the families *Bifidobacteriaceae*, *Lachnospiraceae, Tannerellaceae*, *Mathonobacteriaceae, Barnesiellaceae,* and *Prevotellaceae* were enriched in the QE group; and *Bacteroidaceae, Desulfovibrionaceae, Peptotostretococcaceae,* and *Fusobacteriaceae* were enriched in the Q group, whereas those of *Lactobacillaceae, Veillonellaceae, Ruminococcaceae, Akkermansiaceae,* and *Rikenellaceae* were enriched in the E group compared to the control group. Untargeted metabolomics analyses revealed that Q, E, and QE modified the abundance of several metabolites in prominent pathways including ubiquinone and other terpenoid–quinone biosynthesis, regulation of actin cytoskeleton, insulin secretion, pancreatic secretion, nicotine addiction, and metabolism of xenobiotics by cytochrome P450. Furthermore, key cecal microbiota, significantly correlated with important metabolites, *for example,* (*S*)-equol positively correlated with *Alistipes* and *Chlamydia* in E_vs_C, and negatively correlated with *Olsenella*, *Paraprevotella,* and *Mucispirillum* but, a contrary trend was observed with *Parabacteroides* in QE_vs_C. This study establishes that the synergy of quercetin and vitamin E alters the cecal microbial composition and metabolite profile in aged breeder hens, which lays a foundation for chicken improvement programs.

## Introduction

The gut microbiota plays important biological roles in metabolism, nutrition, physiology, and immunoregulation in both animals and humans (O’Hara and Shanahan, 2006; Konturek et al., 2015). There is a close correlation between the age of the host and its microbiota, thus, during aging, the diversity and richness of the gut microbiota increase (Claesson et al., 2012; Scholtens et al., 2012; Lim et al., 2015), whereas, in old age the composition becomes less diverse and more dynamic (Bischoff, 2016). Furthermore, the intestinal structure and immune barrier integrity are closely correlated with the gut microbiota; hence, it is important to promote gut microbiota composition to improve gut health (Grivennikov et al., 2012). In addition, the physiological performance of broiler breeder hens mostly declines at approximately 65–70 weeks of age (Liu et al., 2018).

Diet is an influential factor that affects the composition, diversity, and function of the gut microbiota in both animals and humans (David et al., 2014; Sonnenburg and Bäckhed, 2016; Zmora et al., 2019). Therefore, age-related dietary changes can affect the composition and function of gut microbiota (Clements and Carding, 2018). Moreover, the use of antibiotics in both animals and humans generates problems, such as antibiotic resistance (Laxminarayan et al., 2013), and reduced diversity of the gut microbiota (Sonnenburg and Sonnenburg, 2019). Hence, developing dietary interventions or possible therapeutic approaches (alternatives to antibiotics) to counteract age-related dysbiosis in aged animals could improve performance (Ghosh et al., 2020). Furthermore, microbial fermentation of dietary supplements produces vital metabolites, such as short-chain fatty acids (SCFAs) of biological significance (Zhu et al., 2019; Chen et al., 2020; Uyanga et al., 2021). Therefore, a negative change in gut microbiota composition and metabolite profile can cause several metabolic and physiological disorders in animals (Sekirov et al., 2010).

The physiological performance of broiler breeder hens mostly declines at approximately 65–70 weeks of age due to age-related degeneration of the small intestine morphological structures, such as villi height, crypt depth, bacterial composition, and organ dysfunction (Liu et al., 2018; Yang et al., 2020).

Vitamin E, a fat-soluble nutrient, has potent antioxidant function with several other biological effects, such as stress alleviation, protection of cells against apoptosis, anti-inflammatory function, and immunoregulation (Jiang, 2014; Wang et al., 2021; Amevor et al., 2022b). Vitamin E intake has been reported to enhance gut microbiota composition and improve gut health in chickens (Choi et al., 2020; Wang et al., 2021); however, there are insufficient data to demonstrate the correlation between *α*-tocopherol (vitamin E) and gut microbiota.

Quercetin, a known flavonoid and prebiotic, exhibits anti-microbial, anti-inflammatory, anti-aging, anti-oxidative, and immunoregulatory effects. Previous studies have indicated that quercetin and other flavonoids alter the gut environment by regulating the gut microbiota composition and its metabolites, as well as intestinal barrier function (Etxeberria et al., 2015; Singh et al., 2019; Dong et al., 2020; Shu et al., 2020).

However, no studies have investigated the effects of the dietary combination of quercetin and vitamin E on the cecal microbiota and its metabolite profile in breeder chickens. Therefore, in the present study, the cecal microbial composition and its metabolites in aged breeder hens fed a combination of quercetin and vitamin E were investigated using 16S rRNA gene sequencing and untargeted metabolomics analysis. Therefore, comparing the differences in the cecal microbiota composition and its potential biochemical metabolites might help us understand how the dietary combination of quercetin and vitamin E alter the gut metabolism, health, and performance of aged breeder hens.

## Materials and Methods

### Experimental Animals, Diets, and Treatments

A total of 400 (65 weeks old) Tianfu broiler breeder hens were randomly allotted to four dietary groups (n = 100 hens each), with 4 replicates per group (*n* = 25 hens each). Four experimental diets were prepared, and each of the groups was fed one of the following four diets: Basal diet (Control) only designated C; Q diet, the basal diet supplemented with dietary quercetin (0.4 g/kg); E diet, the basal diet supplemented with dietary vitamin E (0.2 g/kg); and QE diet, the basal diet supplemented with the combination of dietary quercetin (0.4 g/kg) and vitamin E (0.2 g/kg). The composition and nutritional values of the basal diet for the experiment have been described in our previous study (Amevor et al., 2021a).

Quercetin and vitamin E were supplied by Shaanxi Huike Plant Development Co., Ltd., China (Xian, Shaanxi, China). The purity of the quercetin was determined by high-performance liquid chromatography (purity 95%). The experimental hens were kept in an individual wire cages with the following dimensions: width: 48.8 cm, depth: 38.1 cm, and height: 38.1 cm, under controlled lighting of 16 l:8 D, temperature of 22 ± 1°C and optimal ventilation during the experimental period. The chickens had access to free flow water *ad libitum*. The chickens were managed for a period of 10 weeks. During the experimental period, data on the feed intake and body weight were recorded.

### Sample Collection

At the end of the experimental period (10 weeks), two hens per replicate were randomly selected and euthanized for sample collection. From each of the selected hens, cecal contents were collected aseptically, and to normalize any dissimilarity of the gut microbiota, three samples per each replicate (0.5 g per sample) were mixed and collected (32 cecal samples) into sterilized tubes, and immediately frozen in liquid nitrogen and later stored at −80°C for subsequent microbial DNA extraction.

### DNA Extraction, V4 Region of 16S rDNA Amplicon Pyrosequencing, and Bioinformatics Analysis

#### Extraction of Microbial DNA

Total genomic DNA was extracted from the cecal samples using CTAB method, and the DNA concentration and purity were monitored on 1% agarose gels. The DNA was then diluted to 1 ng/μL using sterile water.

#### 16S rRNA Amplicon Pyrosequencing

16S rRNA genes of the distinct region (V4/16S) were amplified using specific primers (515F: GTGCCAGCMGCCGCGGTAA - 806R: GGACTACHVGGGTWTCTAAT). All PCR were carried out with 15 μl of Phusion® High-Fidelity PCR Master Mix (New England Biolabs); 2 μM of forward and reverse primers, and 10 ng of template DNA. Thermal cycling consisted of initial denaturation at 98°C for 1 min, followed by 30 cycles of denaturation at 98°C for 10 s, annealing at 50°C for 30 s, and elongation at 72°C for 30 s, and finally at 72°C for 5 min. The same volumes of the 1x loading buffer (Catalog No. 10482055; containing SYB green) were mixed with the PCR products, and then electrophoresis was conducted on 2% agarose gel. PCR products were mixed at an equidensity ratio. Thereafter, the mixture of the PCR products was purified using a Qiagen Gel Extraction Kit (Catalog. No. 28704; Qiagen, Germany) according to the manufacturer’s instructions. Sequencing libraries were generated using TruSeq® DNA PCR-Free Sample Preparation Kits (Catalog No. FC-121-3,001; Illumina, United States) following the manufacturer’s instructions. Furthermore, the quality of the library was assessed using a Qubit@ 2.0 Fluorometer (Thermo Scientific) and Agilent Bioanalyzer 2,100 system. Thereafter, the library was sequenced on an Illumina NovaSeq platform at NovoGene Co., Ltd. (Beijing, China) and 250 bp paired-end reads were generated.

#### Sequence Processing and Bioinformatics Analysis

Paired-end reads were assigned to samples based on their unique barcodes and truncated by cutting off the barcode and primer sequences. The paired-end reads were merged using FLASH (V1.2.7; Magoč and Salzberg, 2011) to obtain raw tags. Quality filtering of the raw tags was performed under specific favorable filtering conditions to obtain high-quality clean tags (Bokulich et al., 2013) according to the Quantitative Insights Into Microbial Ecology (QIIME, Version 1.9.1) quality controlled process (Caporaso et al., 2010). The tags were compared with the reference database (Silva database, using the UCHIME Algorithm (Edgar et al., 2011) to detect the chimera sequences, after, the chimera sequences were removed (Haas et al., 2011) to obtain the Effective Tags.

For operational taxonomic units (OTUs) production, sequence analysis was performed using Uparse software (Uparse v7.0.1001; Edgar, 2013). The sequences with ≥97% similarity, were assigned to the same OTUs. The representative sequences for each OTU were screened for further annotation.

Moreover, to study the phylogenetic relationship of the different OTUs, and the differences among the dominant species in different cecal samples per group, multiple sequence alignments were conducted using the MUSCLE software (Version 3.8.31; Edgar, 2004). Thereafter, the OTU abundance information was normalized using a standard sequence number corresponding to the cecal samples with the least sequences. Subsequent analyses of alpha and beta diversities were performed based on the normalized output data obtained.

Alpha diversity indices including Observed-species, Chao1, Shannon, Simpson, ACE, and Good-coverage were calculated with QIIME software (Version 1.9.1) and displayed with R software (Version 2.15.3). In addition, Beta diversity on both weighted and unweighted unifrac was calculated using the QIIME software (Version 1.9.1). Moreover, cluster analysis was followed by principal component analysis (PCA) to reduce the dimensions of the original variables using the *FactoMineR* package and ggplot2 package in R software (Version 2.15.3). Principal Coordinate Analysis (PCA) was performed to obtain the principal coordinates, and visualize complex, multidimensional data. Thereafter, a distance matrix of the weighted or unweighted unifrac among the samples was transformed to a new set of orthogonal axes, by which the maximum variation factor was demonstrated by the first principal coordinate, and the second maximum variation factor was demonstrated by the second principal coordinate. PCA analysis was performed using the WGCNA package, stat packages, and ggplot2 package in R software (Version 2.15.3).

Furthermore, unweighted Pair-group Method with Arithmetic Means (UPGMA) Clustering was performed using QIIME software (Version 1.9.1). Tax4Fun was functionally predicted by the nearest neighbor method based on the minimum 16S rRNA sequence similarity following the procedure outlined by Huang and Liao (2021). Briefly, the 16S rRNA gene sequence of the prokaryotic whole genome was obtained from the Kyoto Encyclopedia of Genes and Genomes (KEGG) database (Kanehisa et al., 2012) using the BLASTN algorithm with a bitscore >1,500 to establish a correlation matrix, and map the KEGG database prokaryotic whole-genome function information annotated by the ultrafast protein classification tool to generate metabolic functional annotations (Meinicke, 2015).

### Untargeted Metabolomics Profiling of the Cecum Content

#### Metabolites Extraction Tissue Sample

The cecal samples were prepared and the supernatant was injected into a liquid-mass spectrometry (LC–MS/MS) system for analysis (Want et al., 2012; Yuan et al., 2012). Furthermore, the ultra-high-performance liquid Chromatography (UHPLC)-MS/MS analyses were performed using a Vanquish UHPLC system (Thermo Fisher, Germany) coupled with an Orbitrap Q Exactive™ HF mass spectrometer (Thermo Fisher, Germany) at Novogene Co. Ltd. (Beijing, China). The cecal samples were then injected onto a Hypersil Gold column (100 × 2.1 mm, 1.9 μm) using a 17 min linear gradient at a flow rate of 0.2 ml/min. The eluents for the positive polarity mode were eluent A (0.1% FA in Water) and eluent B (Methanol). The eluents for the negative polarity mode were eluent A (5 mM ammonium acetate, pH 9.0) and eluent B (Methanol), and the solvent gradient was set as follows: 2% B, 1.5 min; 2–100% B, 12.0 min; 100% B, 14.0 min; 100–2% B, 14.1 min; and 2% B, 17 min. Then, the Q ExactiveTM HF mass spectrometer was operated in positive/negative polarity mode with spray voltage of 3.2 kV, capillary temperature of 320°C, sheath gas flow rate of 40 arb, and aux gasflow rate of 10 arb.

The raw data files generated by UHPLC–MS/MS were processed using Compound Discoverer 3.1 (CD3.1, Thermo Fisher) to perform peak alignment, peak picking, and quantitation for each metabolite.

#### Statistical Analysis

Statistical analyses were performed using statistical R software (Version 2.15.3). Metabolites were annotated using the KEGG database. Principal component analysis (PCA) and Partial least squares discriminant analysis (PLS-DA) were performed using metaX. Univariate analysis (t-test) was used to calculate statistical significance (*p*-value). Metabolites with VIP > 1 and *p* < 0.05 and fold change (FC) ≥ 2 or FC ≤ 0.5 were considered differentially accumulated metabolites (DAMs) in pairwise comparisons among the treatments (Q, E, QE, and C). The functional annotation of the DAMs was conducted in the KEGG database (Kanehisa et al., 2012), and pathways with p < 0.05 were declared significantly enriched pathways (Nguyen et al., 2021).

Pearson’s correlation coefficients were computed and visualized between the selected differential bacterial genera and differential metabolite expression using the *ggplot2* package in R statistical software with a significance threshold set at *p* < 0.05.

## Results

### Impacts of Dietary Q, E, and Q + E on Feed Intake and Body Weight of Aged Hens

**Figure 1:**
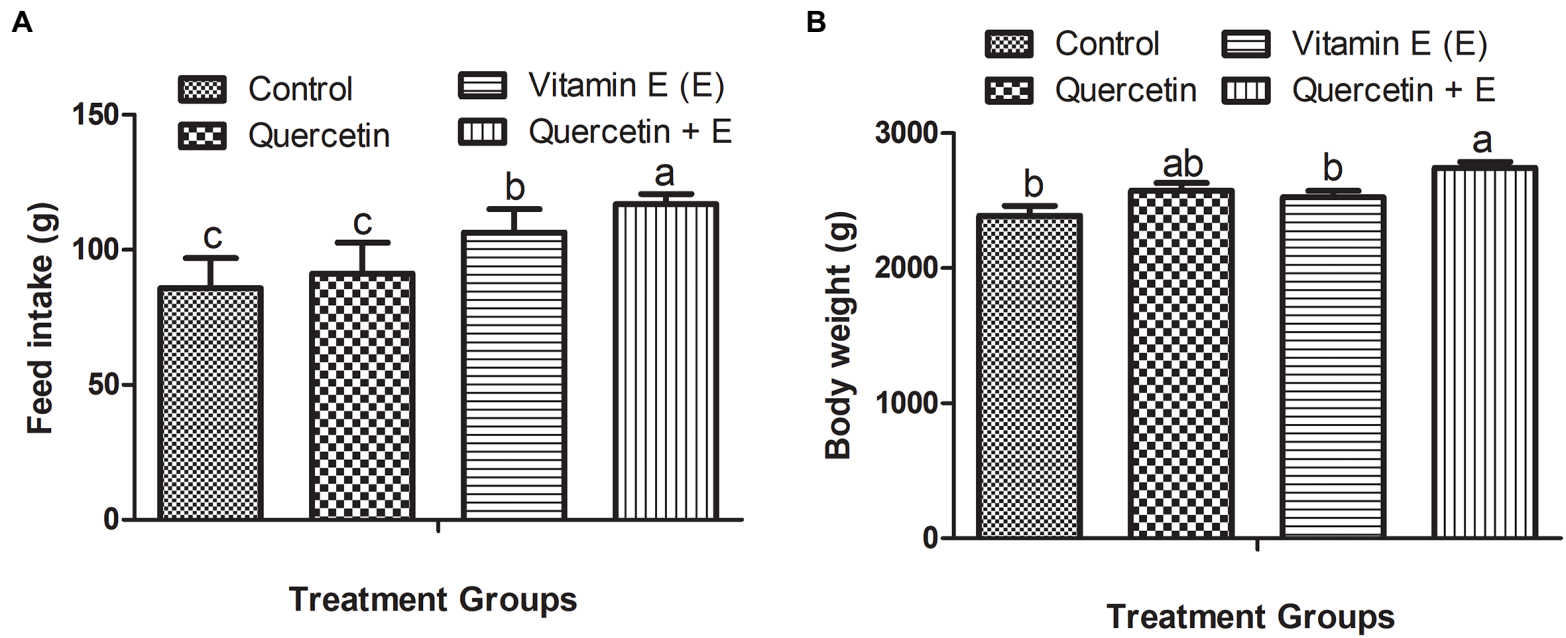
Impacts of dietary quercetin, vitamin E, and the combination of quercetin and vitamin E on the feed intake and body weight of aged broiler breeder hens. Bars without the same letter differed significantly (*p* < 0.05). **(A)** Feed intake; **(B)** body weight.

Figure 1 represents the feed intake and body weight of the aged hens. It was observed that during the 10-week experimental period, Q + E significantly increased the average daily feed intake per hen as compared with the other dietary groups. Moreover, vitamin E also significantly increased feed intake as compared to the control and quercetin dietary groups (Figure 1A, *P* < 0.05). Furthermore, Q + E groups significantly increased the body weight as compared with the control and vitamin E groups (Figure 1B, *P* < 0.05).

### Diversity and Structure of the Cecal Microbiota

The composition and diversity of the cecal microbiota were profiled in the cecum of 8 aged breeder hens per group (C, Q, E, and QE). From this, an average read ± standard deviation of 102316.29 ± 1613.62, 103707.13 ± 1040.30, 103329.50 ± 1662.03, and 104619.38 ± 1371.48, were obtained from the 4 dietary treatments, respectively (Supplementary Table S1). These corresponded to 1,403 distinct operational taxonomic units (OTUs) at the 97% identity level were obtained from all samples; however, after rare OTUs <0.005% of the total OTUs were removed, an average of 924 OTUs were retained for further downstream analyses (Supplementary Table S2).

**Figure 2:**
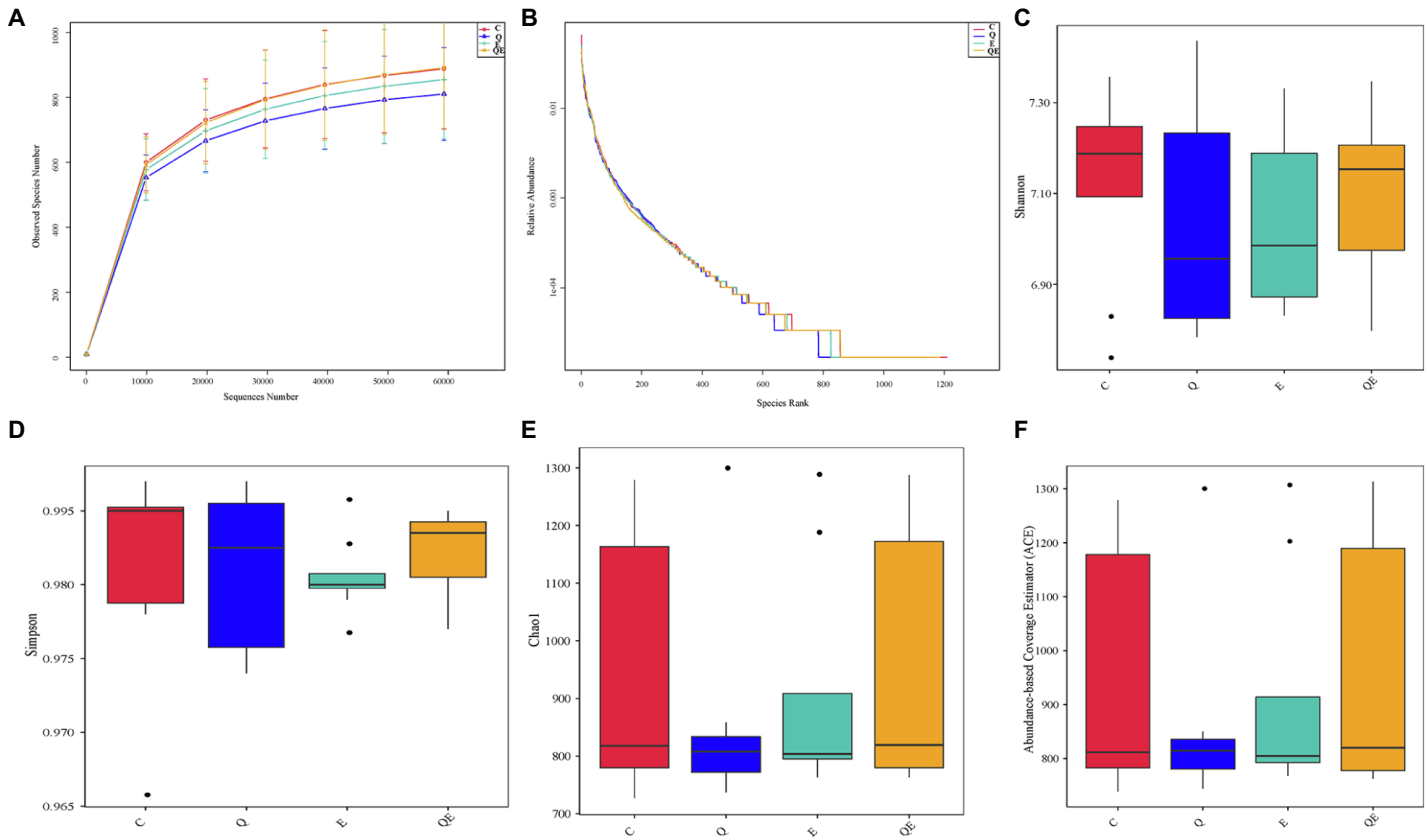
Effects of dietary treatments [(control group (C), quercetin group (Q), vitamin E group (E) and combination of quercetin and vitamin E group (QE)] on cecal microbiota diversity and composition in aged breeder chickens. **(A)** Rarefaction curves, computed at the subsample size of 6,000 per sample, exhibit the effects of sequencing efforts on the observed number of OTUs at 97% sequence similarity. The error bars represent standard deviation of the eight replicates. **(B)** Abundance curve of species identified. **(C)** Shannon diversity index boxplot. **(D)** Simpson diversity index plot. **(E)** Chao1 diversity index boxplot. **(F)** Abundance-based coverage estimator (ACE) diversity index boxplot.

The curves for the observed OTUs and species rank (Figures 2A,B) approached a plateau, suggesting that the sequencing depth was adequate for the coverage of all OTUs present in the cecal samples. Alpha diversity indices, such as Shannon, Simpson, Chao1, and abundance-based coverage estimator (ACE; Figures 2C–F) did not differ statistically among the 4 dietary groups. These results corroborate the results of principal component analysis (PCA) that, 32 cecal samples showed no difference in the community phylotype structure (Supplementary Figure S1), indicating that the aged breeder chickens had similar microbial communities in the cecum among the treatments. On the other hand, the first two principal components could explain 43.99% variation in the bacterial diversity in the cecum.

**Figure 3:**
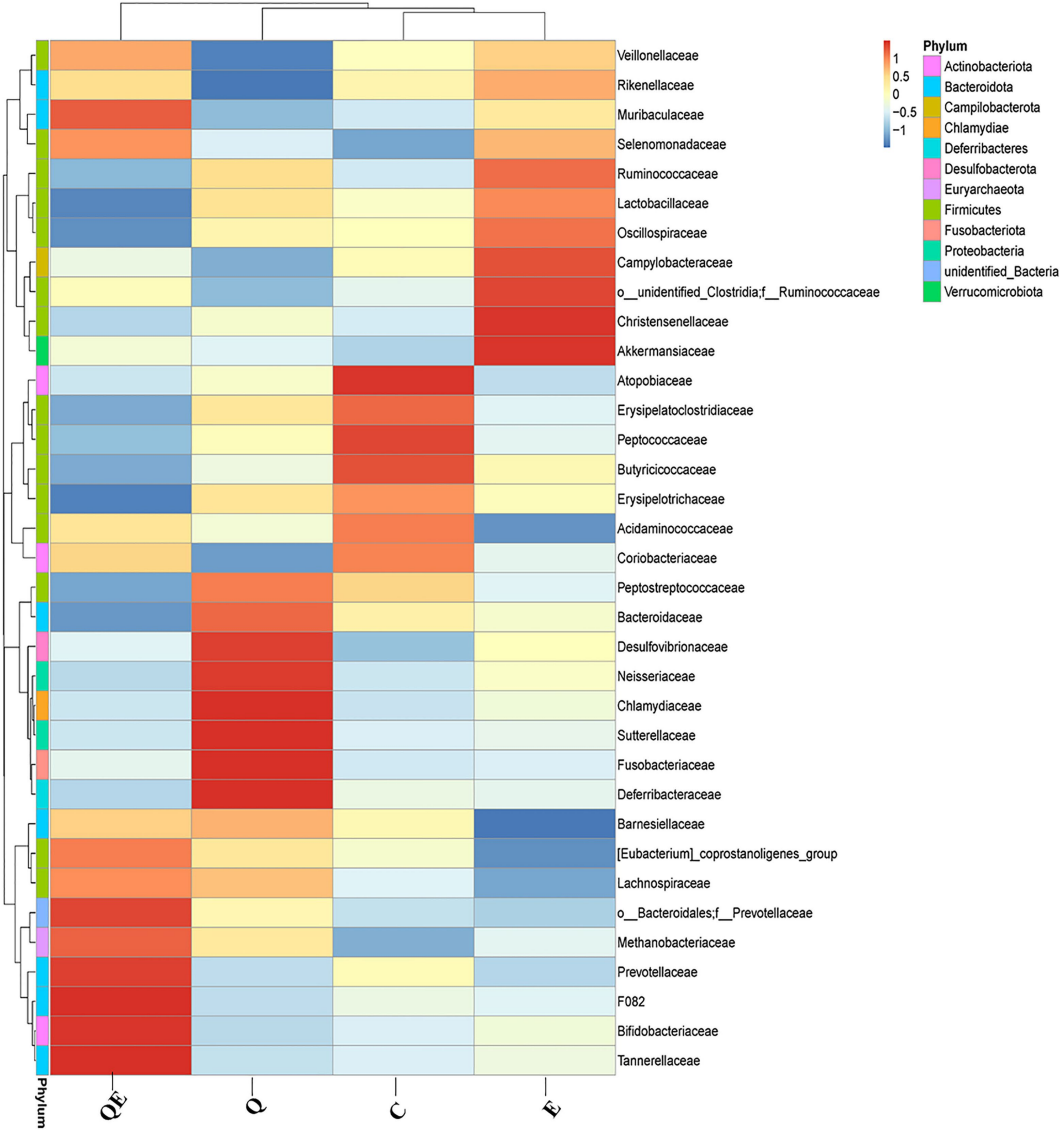
Cecal microbiota composition and diversity at the phylum and family levels among the aged breeder chickens fed with quercetin (Q), vitamin E (E), and QE compared with the control group (C) by phylogenetic relationship and weighted unifrac distance between OTUs. The legend for color intensity of family and phylum are given on the right-hand side of the heatmap clustering figure.

In contrast, the weighted unifrac distance between OTUs clustered the 4 dietary treatments into 2 major clusters: Cluster I with only the QE group, while Cluster II consisted of C, Q, and E groups (Supplementary Figure S2). At the phylum level, the top 11 groups of the cecal microbiota were largely dominated by *Bacteroidota*, *Firmicutes,* and *Actinobacteriota* (Supplementary Figure S2). At the family level, Figure 3 shows a contrasting pattern of abundance of the various microbiota identified among the 4 dietary groups, suggesting that the inclusion of either vitamin E, quercetin, or a combination of quercetin and vitamin E (quercetin + vitamin E) alters the family level in the cecum in contrast to the control group. For instance, the relative abundance of the families *Bifidobacteriaceae*, *Lachnospiraceae, Tannerellaceae*, *Mathonobacteriaceae, Barnesiellaceae,* and *Prevotellaceae* was enriched in the cecal microbiota of the QE group; that of *Bacteroidaceae, Desulfovibrionaceae, Peptotostretococcaceae,* and *Fusobacteriaceae* was enriched in the cecal microbiota of the Q group, whereas, the relative abundance of the families *Lactobacillaceae, Veillonellaceae, Ruminococcaceae, Akkermansiaceae,* and *Rikenellaceae* was enriched in the cecal microbiota of the E group, compared with the control group (Figure 3).

**Figure 4:**
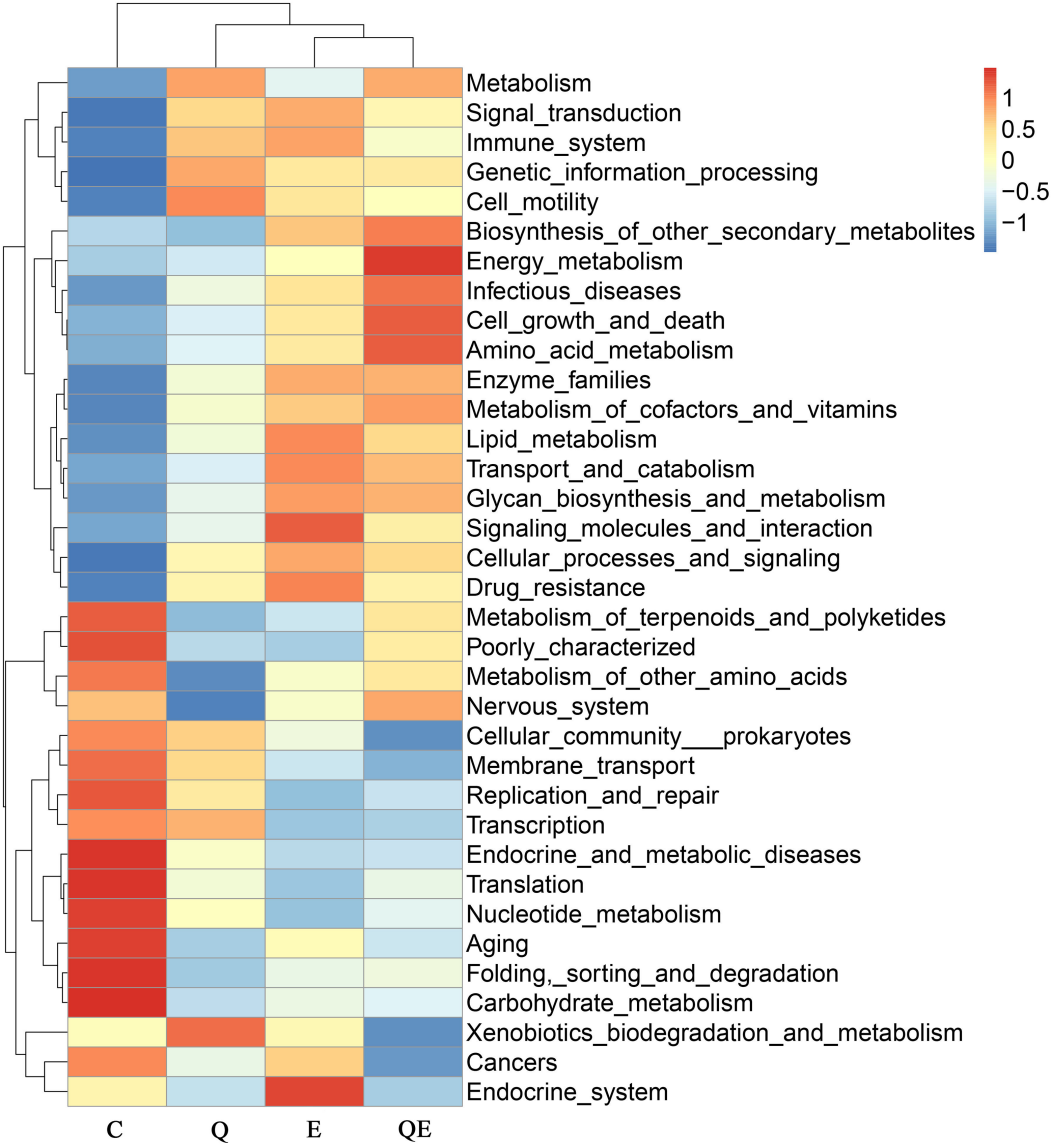
Heatmap clustering of topmost 35 Kyoto Encyclopedia Genes and Genomes of the genus microbiota identified in the cecum of the aged breeder chickens fed with dietary quercetin (Q), vitamin E (E), and QE compared with the control group (C).

Similarly, the top 35 KEGG pathway enrichment analyses of the families (Figure 4) showed trends similar to those of the family levels presented in Figure 3. Among the 35 topmost pathways, carbohydrate metabolism increased in abundance in the C group compared to the Q, E, and QE groups (Figure 4; Supplementary Table S3). In addition, the control group had the highest family enrichment in the metabolism of terpenoids and polyketides, metabolism of other amino acids, nervous system, cellular community prokaryotes, membrane transport, replication and repair, transcription, endocrine and metabolic diseases, translation, nucleotide metabolism, aging, folding, sorting and degradation (Figure 4). These results suggest that the aged breeder hens in the control group spent more energy on maintenance other than reproduction and health relative to the birds fed with dietary Q, E, and QE.

In contrast, QE enhanced the abundance of genes involved in metabolism, signal transduction, the immune system, genetic information processing, cell motility, biosynthesis of other secondary metabolites, energy metabolism, infectious diseases, cell growth and death, amino acid metabolism, enzyme families, metabolism of cofactors and vitamins, lipid metabolism, transport and catabolism, glycan biosynthesis and metabolism, signaling molecules and interaction, cellular processes and signaling, and drug resistance (Figure 4; Supplementary Table S3).

### Effects of Dietary Quercetin, Vitamin E, and Combination of Quercetin and Vitamin E on Cecal Metabolites

To profile and select differential metabolites for biomarker development in our attempt to make aged breeder chickens productive, healthy, and prolific, we further conducted non-targeted metabolome profiling of the cecum contents in the 32 chickens used in this study *via* UHPLC combined with LC–MS/MS technology. We detected 1,896 metabolites comprising 1,210 and 686 metabolites at the positive and negative nodes, respectively (Supplementary Tables S4A,B). Owing to the large volume of data, we performed further analysis on the 1,210 metabolites detected in the positive node.

**Figure 5:**
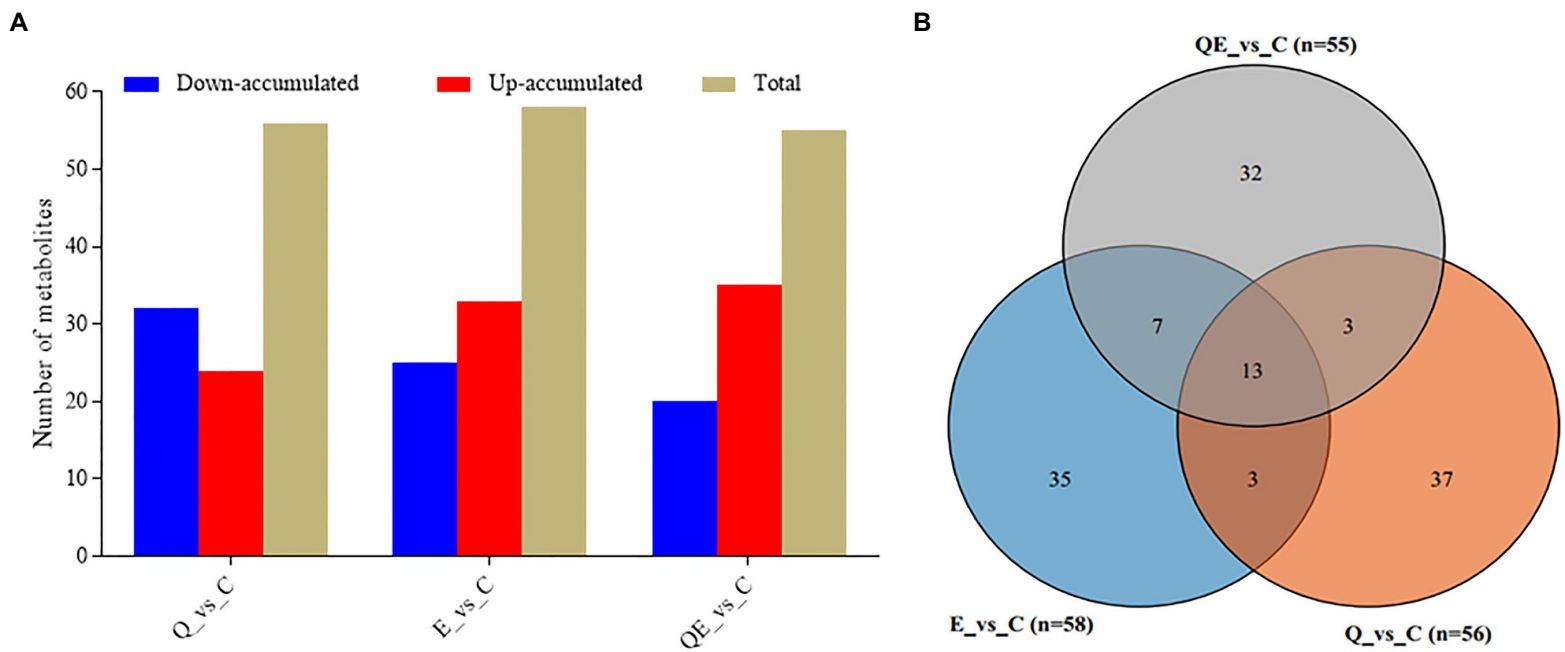
Differentially accumulated metabolites (DAMs) detected in the cecum of the aged breeder chickens fed with dietary quercetin (Q), vitamin E (E), and QE compared with the control group (C). **(A)** Total number of DAMs and their accumulation levels. **(B)** Venn diagram of DAMs detected in the C group against either Q, E, or QE group. The partial least square discriminant analysis of log2 fold change ≥1 and variable importance in projection ≥1 were used to screen for DAMs.

To screen for differentially accumulated metabolites (DAMs) among the groups, we applied partial least squares-discriminant analysis (PLS-DA; Supplementary Figure S3) together with the variable importance of the projection VIP > 1 and *p* < 0.05 and fold change (FC) ≥ 2 or FC ≤ 0.5. A total of 58, 56, and 55 DAMs were detected in E_vs_C, Q_vs_C, and QE_vs_C, respectively (Figures 5A,B; Supplementary Table S5). The supplementation of vitamin E or quercetin + vitamin E largely induced the upregulation of more metabolites relative to the control group (Figure 5A; Supplementary Table S5). For example, DL-Carnitine accumulated 6.23, 3.48, and 2.64 times higher in the cecum of aged breeder chickens in the E, QE, and Q groups, respectively than that in the control group (Supplementary Table S5). This compound is well known to improve broiler growth, productivity, carcass characteristics, and immunity by facilitating fatty acid β-oxidation, and decreasing esterification reactions and triacylglycerol storage in adipose tissue (Ghoreyshi et al., 2019).

**Figure 6:**
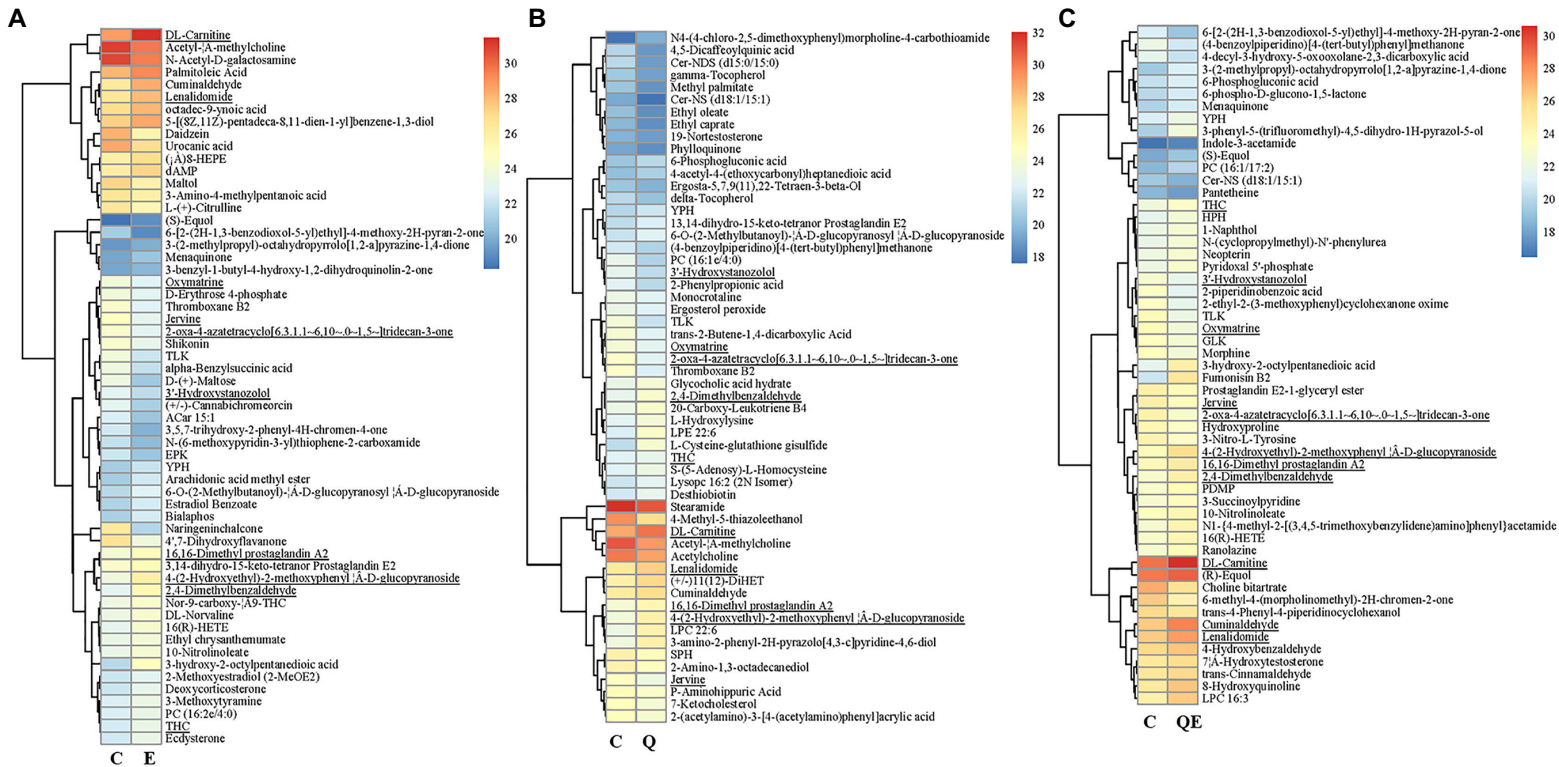
Heatmap clustering of differentially accumulated metabolites (DAMs) and their transformed ion intensities (log2) detected in the cecum of the aged breeder chickens fed with quercetin (Q), vitamin E (E), or QE compared with the control group in pairwise comparison (C). **(A)** E_vs_C. b. Q_vs_C. c. QE_vs_C. The partial least square discriminant analysis of log2 fold change ≥1 and variable importance in projection ≥1 were used to screen for DAMs. The underline compounds in **(A–C)** represent those detected concurrently among the three pairwise groups. Deoxyadenosine monophosphate (dAMP).

Another typical compound, cuminaldehyde, was reported to exhibit beneficial effects against various diseases and improve egg production, egg quality, hatchability traits, and blood serum mineral content in laying quails (Şimsek et al., 2015; Goel et al., 2020), and this compound increased in accumulation in the E, Q, and QE groups (thus, 1.90, 1.60, and 1.83 times higher) than that of the C group (Figure 6; Supplementary Table S5).

Among the DAMs detected, 13 were detected concurrently among the 3 pairwise groups (Figure 5b,6), suggesting that either of the 4 groups had some metabolites in common, although there were several metabolites that were unique to some pairwise groups (Figure 5B). This was more evident from the clustering of the 58, 56, and 55 DAMs in E_vs_C, Q_vs_C, and QE_vs_C, respectively (Figure 6).

**Figure 7:**
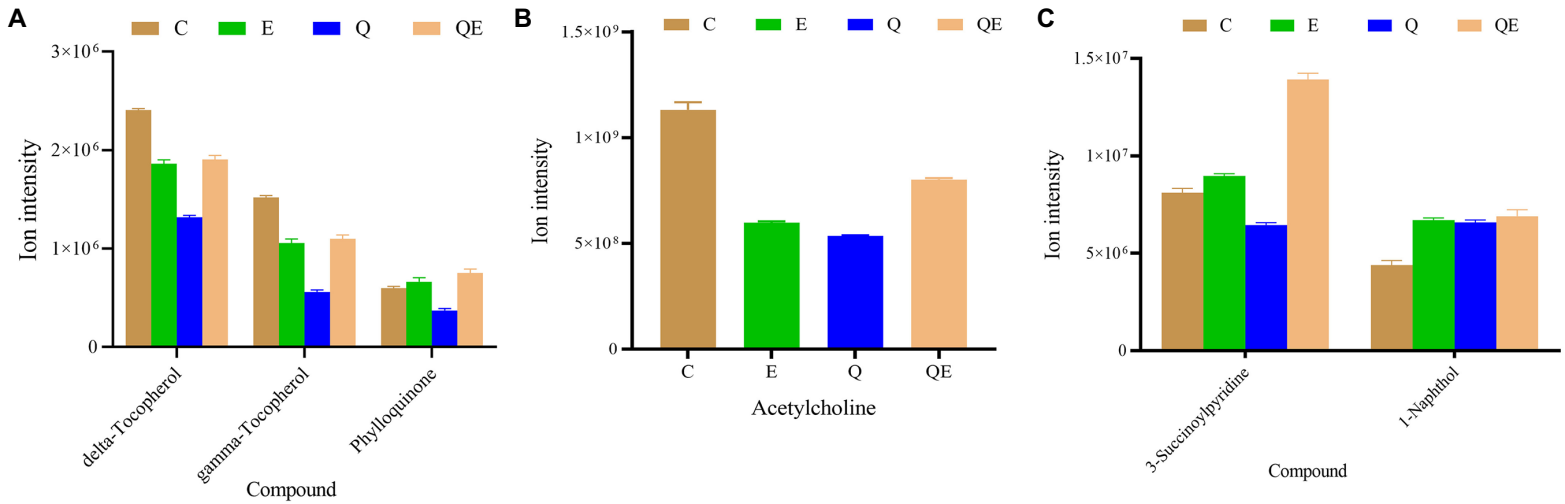
Ion intensities of metabolites involved in differentially expressed pathways by Kyoto Encyclopedia of Genes and Genomes among the aged breeder chickens (control group (C), quercetin group (Q), vitamin E group (E), and QE). **(A)** Ubiquinone and other terpenoid–quinone biosynthesis (delta-Tocopherol, gamma-Tocopherol, and Phylloquinone). **(B)** Regulation of actin cytoskeleton, Insulin secretion, Pancreatic secretion, and Nicotine addiction (Acetylcholine). **(C)** Metabolism of xenobiotics by cytochrome P450 (3-Succinoylpyridine and 1-Naphthol).

We further subjected the DAMs detected in the pairwise groups to KEGG pathway enrichment analyses resulting in 45 unique enriched pathways (Supplementary Figure S4; Supplementary Table S6). Surprisingly, no pathway was significantly enriched in E_vs_C (*p* > 0.05), but in the case of Q_vs_C and QE_vs_C, 5 pathways (ubiquinone and other terpenoid–quinone biosynthesis, regulation of actin cytoskeleton, insulin secretion, pancreatic secretion, and nicotine addiction) and 1 pathway (metabolism of xenobiotics by cytochrome P450) were significantly enriched (*p* < 0.05). We focused on these pathways to identify the metabolites and their ion intensities which are presented in the histogram (Figure 7). We found that Phylloquinone accumulated higher in the E and QE groups than in the C group (Figure 7A). These results suggest a modulating role of this compound in determining the performance of aged breeder chickens.

In addition, E and QE reduced the abundance of acetylcholine (Figure 7B); however, the accumulation of two main metabolites including 3-Succinoylpyridine and 1-Naphthol, that are involved in the metabolism of xenobiotics by cytochrome P450, was increased in the E or QE group compared to the C group (Figure 7C).

**Figure 8:**
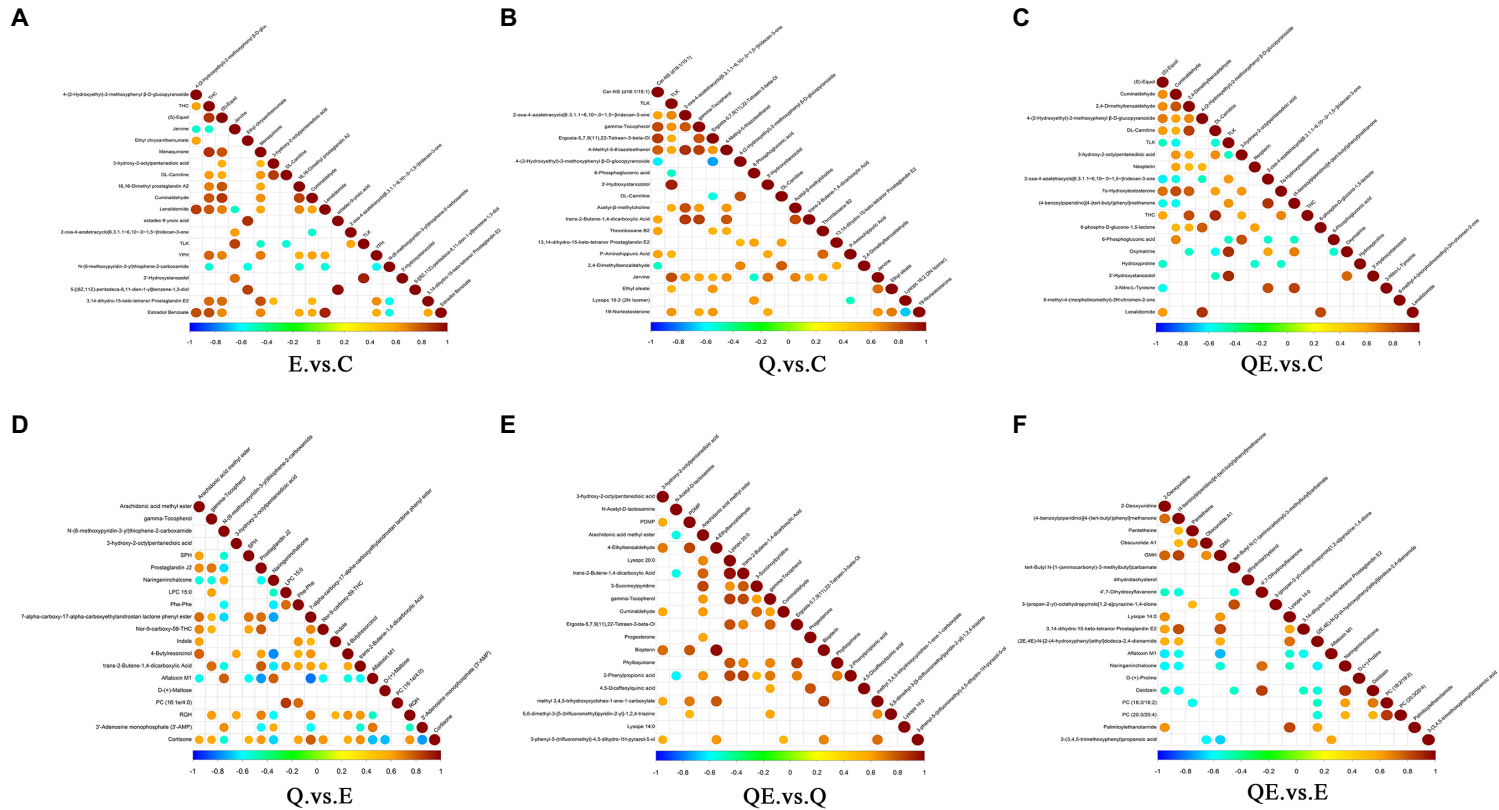
Interaction of differential metabolites among the dietary groups. The highest correlation is 1, which is a complete positive correlation (red), and the lowest correlation is −1, which is a complete negative correlation (blue). The part without color indicates *p* > 0.05. The figure shows the Correlations of the top 20 differential metabolites sorted by value of *p* from small to large. The upper frame is positive ion mode, the lower frame is negative ion mode. **(A)** E_vs_C. **(B)** Q_vs_C. **(C)** QE_vs_C. **(D)** Q_vs_E. **(E)** QE_vs_Q. **(F)** QE_vs_E.

The correlation analysis of differential metabolites among the dietary groups was performed. The purpose of the differential metabolite correlation analysis is to check the consistency of each metabolite and metabolite change trends among the dietary groups and to analyze the correlation between each metabolite by calculating Pearson’s correlation coefficient between all metabolites. We observed a synergistic or mutually exclusive relationship between different metabolites [Figure 8 (A) E_vs_C. (B) Q_vs_C. (C) QE_vs_C. (D) Q_vs_E. (E) QE_vs_Q. (F) QE_vs_E]. Figure 8 showed the top 20 differential metabolites between the dietary groups. For example, certain type of metabolites have the same trend of change, which was positively correlated, whereas others have negative correlation. Thus, when the linear relationship between the two metabolites is enhanced, it tends to 1 for positive correlation and − 1 for negative correlation. Several important differential metabolites, such as ((s)-Equol, DL-carnitine, cuminaldehyde, and Estradiol Benzoate), (DL-carnitine, gamma-Tocopherol, etc.), ((s)-Equol, cuminaldehyde, DL-carnitine, etc.), (gamma-Tocopherol, Arachidonic acid methyl ester, etc.), (gamma-Tocopherol, cuminaldehyde, phylloquinone, 2-phenylpropionic acid, Lysope, Arachidonic acid methyl ester, etc.), and (Lysope, Daidzein, Propanoic acid, D-(+)-Proline, Dihydroxyflavanone, etc.) were highly expressed between the E_vs_C, Q_vs_C, QE_vs_C, Q_vs_E, QE_vs_Q, and QE_vs_E groups, respectively.

**Figure 9:**
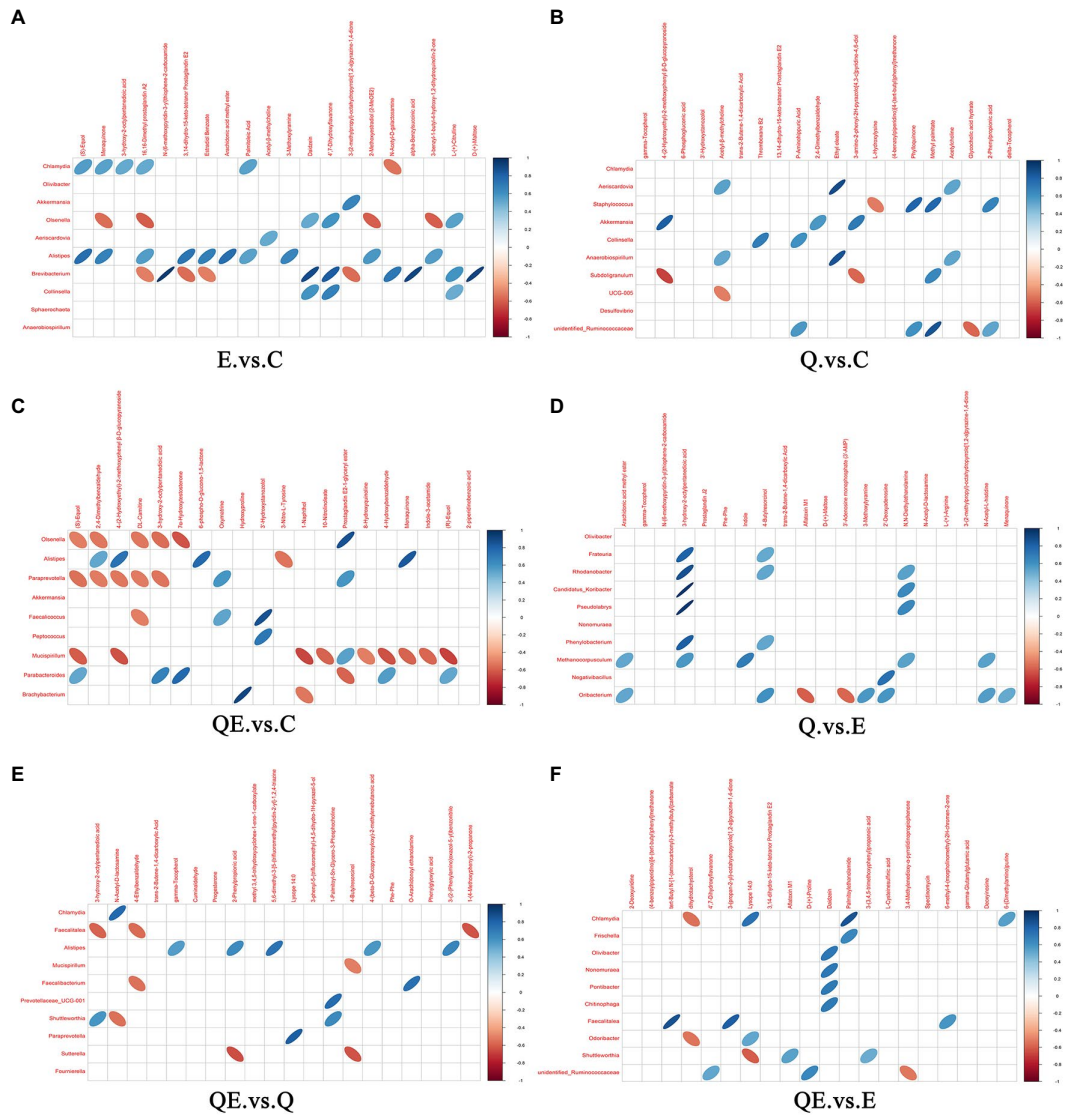
Correlation between microbiota and metabolites in the cecum of aged breeder hens fed quercetin (Q) or vitamin E (E) or QE compared with the control group (C) in a pairwise comparison. **(A)** E_vs_C. **(B)** Q_vs_C. **(C)** QE_vs_C. **(D)** Q_vs_E. **(E)** QE_vs_Q. **(F)** QE_vs_E. The upper frame represents the negative ion mode, and the lower frame represent the positive ion mode. Blue means positive correlation, red means negative correlation, and blank means not statistically significant (*p* > 0.05). Intersection of microbiota and metabolites with oval shape object indicates significant correlation coefficient (*p* < 0.05) and those with no oval object (shape) indicate no significant correlation (*p* > 0.05).

Furthermore, we performed Pearson’s correlation analysis between selected differential metabolites and differential microbiota to identify the metabolites that could be used to increase or decrease the abundance of some microbiota at the genus level in the cecum of aged breeder hens (Figures 9A–F). *Alistipes* which has been reported as one of the dominant species in the cecum of birds (Pandit et al., 2018; Rychlik, 2020) was positively correlated with 10 metabolites in E_vs_C, and 4 metabolites in QE_vs_C (Figures 9A,C). One major metabolite detected in E_vs_C and QE_vs_C, (*S*)-equol was positively correlated with *Chlamydia* and *Alistipes* in E_vs_C (Figure 9A) and negatively correlated with *Olsenella*, *Paraprevotella,* and *Mucispirillum,* but contrary trends were observed for *Parabacteroides* in QE_vs_C (Figure 9C). In addition, it was observed that *Oribacterium* was negatively correlated with Aflatoxin M1, whereas it was positively correlated with 3-Methoxytyramine, 2-Deoxyadenosine, N-acetyl-L-histidine, and Menaquinone in Q_vs_E (Figure 9D). Moreover, 3-Hydroxy-2-octylpentanedioic acid positively correlated with six differential bacterial genera *Frateuria*, *Rhodanobacter*, *Candidatu-koribacter*, *Pseudolabrys*, *Phenylobacterium*, and *Methanocorpusculum*.

## Discussion

In animals, the gut is home to several microbiota closely associated with metabolism, reproduction, and health. However, during the late stages of life, microbiota composition becomes less diverse and dynamic (Bischoff, 2016). Many studies have reported that diets alter the composition, diversity, and function of gut microbiota, as well as its metabolites in animals (Sonnenburg and Bäckhed, 2016; Zmora et al., 2019). For instance, flavonoids, vitamins, and amino acids have been reported to improve the gut microbiota composition and health of animals (Etxeberria et al., 2015; Choi et al., 2020; Dong et al., 2020; Shu et al., 2020; Uyanga et al., 2021; Wang et al., 2021). In our previous study, we found that dietary supplementation with quercetin and vitamin E improved egg production, feed efficiency, egg quality, immunity, follicle development, liver function, and reproductive organ function in aged laying hens (Amevor et al., 2021a,b), which was consistent with other studies (Liu et al., 2014; Abedi et al., 2017; Dalia et al., 2018; Yang et al., 2021).

Dietary quercetin and vitamin E may promote performance in aged breeder hens by altering the gut microbiota, thereby improving nutrient absorption in the small intestine, stimulating the production and activity of digestive enzymes, and stimulating digestive and physiological metabolism (Dong et al., 2020; Uyanga et al., 2021; Wang et al., 2021; Amevor et al., 2022a). It was observed in this present study that the supplementation of the combination of dietary quercetin and vitamin E improved the palatability of the diet, hence, resulted in improving the feed intake. The results of this study showed that microbial composition revealed no significant differences in the community phylotype structure and alpha diversity of the cecal microbiota among the dietary groups. These findings were consistent with the study by Dong et al. (2020), who reported that dietary quercetin supplementation in broiler chickens fed with oxidized oil showed no significant differences among the alpha diversity indices (Dong et al., 2020), as well as the study by Chen et al. (2020), who reported that plant essential oils supplementation had no effect on cecal microbial alpha diversity in broiler chickens. Moreover, we observed more variation in the cecal microbiota in the aged hens among the groups, which was consistent with previous studies in Cynomolgus monkeys and humans, indicating that, the gut microbiota of adult groups of animals or humans shows more variation than that in the younger group (Lan et al., 2013; Duan et al., 2019). In addition, Pourabedin et al. (2015) reported that overall microbial diversity was not affected by dietary treatment with either xylo-oligosaccharides or virginiamycin in chickens. This finding suggest that the diversity of the gut microbiota may change dynamically in aged chickens.

In the present study, *Bacteroidota*, *Firmicutes,* and *Actinobacteriota* were dominant among the groups at the phylum level. This finding was consistent with previous studies in chickens (Pourabedin et al., 2015; Huang et al., 2018; Pandit et al., 2018; Khan et al., 2020), and the results of Chen et al. (2020), who reported a decreased relative abundance of the phylum *Firmicutes*, but an increased relative abundance of *Bacteroidota* in birds fed with plant essential oils. The differences observed between the present study and previous studies may be attributed to differences in chicken breeds, age of chickens, feed composition, and feeding patterns (Zeng et al., 2015).

QE and Q increased the abundance of *Firmicutes* ([*Eubacterium]_coprostanoligenes_group* and *Lachnospiraceae*) compared with the control group, which is consistent with the findings of Zhao et al. (2021) who found that quercetin reversed monosodium glutamate-induced abdominal obesity as a result of hypothalamic damage which causes health problems by elevating the abundance of *Firmicutes*. Additionally, QE decreased the abundance of *Ruminicoccaceae,* whereas, QE/Q/E reduced the abundance of *Erysipelatociostridiaceae, Peptococcaceae, Butyricicoccaceae*, *Erysipeltrichaceae,* and *Acidaminococcaceae.* This result was consistent with the work by Lan et al. (2021), who reported that quercetin partially abrogates gut microbiota disorders by increasing *Lactobacillus* abundance and decreasing *Ruminicoccaceae* abundance. Variations in the abundance of some prominent families may be the basis for the enhanced reproductive performance, immunity, and gut function observed in our earlier studies (Amevor et al., 2021a,b).

Two probiotic strains *Bifidobacterium breve* JM1192 and *B. infantis* BL2416 have been reported to improve body weight gain and prevent the deleterious effects and mortality induced by *Salmonella typhimurium* infection in chicks through different mechanisms, such as competitive exclusion and promotion of cytokine release (El-Sharkawy et al., 2020); therefore, the increased abundance of *Bifidobacteriaceae* in the QE dietary treatment group is not surprising. Supplementation with QE increased the relative abundance of *Lachnospiraceae,* which is involved in butyrate production, and has the capability to form conjugated linoleic acid from linoleic acid (Neyrinck et al., 2012). In addition, *Lachnospiraceae* was reported to play important roles in dietary glycan foraging, beneficial metabolite production, and immunity, as well as promoting neurodevelopment in animals (Oliphant et al., 2021). Moreover, *Rikenellaceae,* which is enhanced by vitamin E supplementation in this study, is responsible for producing organic acids, such as propionic and succinic acids by the fermentation of glucose, melibiose, mannose, and lactose, to form iso-methyl branched-chain fatty acids or long-chain saturated acids (Graf, 2014). The biological roles of these bacteria promote animal performance (Neyrinck et al., 2012; Graf, 2014).

Among the numerous pathways enriched according to our KEGG functional analysis, the microbiota in the cecum of the QE dietary treatment group increased the biosynthesis of several metabolic compounds, including amino acids, lipids, and glycans compared with the control group, most of which were identified in the study by Huang et al. (2018).

In addition, nucleotides are semi-essential nutrients, and under conditions of rapid growth, stress, and disease lead to insufficient synthesis based on the capacity of the animal (Kruger and Werf, 2018). Interestingly, the inclusion of either Q, E, or QE in the diet of aged breeder hens was uniquely enriched in nucleotide metabolism; thus, inclusion of feed supplements (Q, E, or QE) may have enhanced the synthesis of nucleotides. Animals with enhanced nucleotide synthesis are reported to have improved productivity in terms of average daily gain and feed conversion ratio, improved immune cells and antibodies, reduced impact of pathogenic infections, improved gut development and integrity, and improved meat quality (Kruger and Werf, 2018; Wu et al., 2018). In addition, the activities of reproductive hormones, such as estrogen and progesterone, which are involved in follicle development in chickens are coordinated by signal transduction (Brady et al., 2021; Chen et al., 2021) which is increased in enrichment among the Q, E, and QE groups compared to the control group.

Biomarkers are biological characteristics that are objectively quantified and evaluated as indicators of normal or abnormal biological processes, pathological processes, or pharmacological responses to therapeutic intervention (Stowasser, 2016). In recent years, with advancements in technology, several biomarkers have been developed and deployed in the poultry industry (Ducatelle et al., 2018; Baxter et al., 2019). For instance, D-lactate is a metabolite produced by intestinal bacteria and has been deployed as a serum biomarker of intestinal permeability in chickens (Lei et al., 2013). With this in mind, we profiled metabolites in the cecum of 32 aged breeder hens fed with dietary C, Q, E, or QE.

The inclusion of QE and vitamin E increased the abundance of essential metabolites, such as (*S*)-equol, which has been reported to enhance chicken performance. For example, (*S*)-equol, a major gut metabolite of daidzein (isoflavone-derived metabolite), has shown antioxidant, estrogenic, unique anti-androgenic, and anti-cancer activities, as well as cardiovascular protective properties (Li et al., 2018). Strikingly, QE dietary treatment exclusively increased the abundance of 8-Hydroxyquinoline 2.76 fold change higher to that in the control group. This compound has been reported to have a wide range of antimicrobial properties, including antibacterial, antiviral, and anti-parasitic effects (Prachayasittikul et al., 2013). This compound and others, such as Ranolazine, 3-phenyl-5-(trifluoromethyl)-4,5-dihydro-1H-pyrazol-5-ol, Fumonisin B2, and many more presented in Supplementary Table S5C, may be the basis for improved performance, organ characteristics, and egg quality in QE diet-treated aged breeder hens (Prachayasittikul et al., 2013; Tardieu et al., 2019).

In contrast, QE decreased the accumulation of metabolites, such as pantothenic acid, Morphine, Prostaglandin E2-1-glyceryl ester, Hydroxyproline, Choline bitartrate, and 3-Nitro-L-Tyrosine. Among these metabolites, 3-Nitro-L-Tyrosine, a derivative of Tyrosine which is involved in Tryptophan metabolism and is further catabolized to the neurotransmitters adrenalin, noradrenalin and dopamine is associated with modulation of feather pecking in chickens (Birkl et al., 2019), suggesting that the synergy of QE may influence egg production, egg quality, body conformation, reproductive organ function, gut functioning, and immunity in chickens.

To identify the biosynthetic pathways, we further searched for differentially enriched pathways *via* KEGG analysis among the three pairwise groups obtained from E, Q, QE, and C. One such prominent pathway was ubiquinone and other terpenoid–quinone biosynthesis pathways with delta- and gamma-tocopherol, and Phylloquinone. Surprisingly, delta- and gamma-tocopherol have been implicated in enhancing egg yolk quality and health status (Hansen et al., 2015; Skřivan et al., 2020). These two pathways were decreased in the Q treatment group, suggesting that inclusion of only quercetin may affect the bioavailability of vitamin E derivatives, thus, there is a need to use quercetin together with a vitamin E in diet supplementation. Moreover, the bioavailability of vitamin K derivative (Phylloquinone) as a result of supplementation with either vitamin E or QE was evidenced in the present study. The supplementation of E and QE promoted the abundance of 3-Succinoylpyridine and 1-Naphthol, which were reported to be involved in the metabolism of xenobiotics by cytochrome P450. Moreover, cytochrome P450 is implicated as the key enzyme for the metabolism or biotransformation of T-2 toxin to 3’OH-T-2 in chickens. This mechanism promotes chicken performance (Shang et al., 2013). These findings are consistent with those reported by Liu et al. (2021), who stated that dietary yeast culture supplementation, improves the biosynthesis of glutathione metabolism, lipopolysaccharide proteins, and ubiquinone and other terpenoid–quinone metabolic pathways which are prominent pathways associated with immune function, egg production, and reproductive efficiency in aged breeder hens (Liu et al., 2021). Therefore, delta- and gamma-tocopherol, Phylloquinone, Acetylcholine, 3-Succinoylpyridine, and 1-Naphthol were validated and targeted for biosynthetic metabolic pathway engineering to improve the performance, gut health, and egg quality of aged breeder chickens.

The bacterial genera most commonly used as probiotics include *Bacillus, Lactobacillus, Enterococcus, Bifidobacterium,* and *Streptococcus* and which influence intestinal function and performance. For instance, they modulate the host immune system, provide energy through microbial metabolites in host microbiota cross talk *via* short-chain fatty acid production, and influence the intestinal structure, integrity and function (Khan et al., 2020). For example, (*S*)-equol showed contrasting correlations with different microbiota in the current study, confirming that there is cross-talk between microbiota and metabolites in the cecum of aged breeder chickens. Therefore, the results presented in this study can be validated and used to discover biomarkers and their use to facilitate layer breeding and genetic improvement programs.

## Conclusion

In general, dietary supplementation with a combination of quercetin and vitamin E, elevated the relative abundance of the genera *Bifidobacteriaceae*, *Lachnospiraceae, Tannerellaceae*, *Mathonobacteriaceae, Barnesiellaceae,* and *Prevotellaceae* and decreased the abundance of the genera *Chlamydiaceae* and *Campylobacteraceae* in the cecum of the aged hens compared to the other groups. Many key metabolites (DL-Camitine, Cuminaldehyde, Lenalidomide, and (R)-Equol) and prominent KEGG pathways (Phylloquinone, 3-Succinoylpyridine, and 1-Naphthol, etc.) responsible for chicken productivity were enriched in the cecum of hens in the QE group. In addition, many microbiota and metabolites were significantly correlated. The findings from this study provide valuable information for understanding the mechanisms by which QE promotes productivity in aged breeder hens by altering the gut microbiota and metabolite profile, providing information on developing natural, effective, and safe dietary alternatives to antibiotics, and finally providing information on biomarker discovery and validation that facilitate layer breeding and genetic improvement programs.

## Data Availability Statement

The datasets presented in this study can be found in online repositories. The names of the repository/repositories and accession number(s) can be found at: https://www.ncbi.nlm.nih.gov/bioproject/PRJNA789878.

## Ethics Statement

The animal study was reviewed and approved by Institutional Animal Care and Use Committee of the Sichuan Agricultural University, Chengdu, China, under permit number 2019502005 (Chengdu, China).

## Author Contributions

FA, XZ, and GS designed and conceived this study. FA, ZC, JF, and ZN conducted the experiments. FA, ZC, XiaxD, DX, XDe, WS, YW, XC, JF, JH, and YT collected the samples and performed the analysis of samples. FA, XZ, GS, ZC, BK, DL, YW, and YZ analyzed the data. FA, XZ, GS, CZ, and XiaxD wrote the manuscript. FA, XZ, QZ, DL, YW, BK, XiaoD, and FA revised and edited the manuscript. All authors contributed to the article and approved the submitted version.

## Funding

The authors thank the Science and Technology Innovation and Entrepreneurship Seedling Project of the Sichuan Science and Technology Program (2020JDRC0104), the Key Research and Development Plan of the Department of Science and Technology of Tibet Autonomous Region (XZ202101ZY0002N), the Local Projects Guided by the Central Government from Razi County, Tibet Autonomous Region, the Projects Funded by the Central Government to Guide Local Scientific and Technological Development from Guizhou province [QIANKEZHONGYINDI(2021)4003], and Bazhong Municipal Government and University Cooperation Project Breeding, Development and Healthy Raising of Meihua chicken in Bazhong for funding this work.

## Conflict of Interest

The authors declare that the research was conducted in the absence of any commercial or financial relationships that could be construed as a potential conflict of interest.

## Publisher’s Note

All claims expressed in this article are solely those of the authors and do not necessarily represent those of their affiliated organizations, or those of the publisher, the editors and the reviewers. Any product that may be evaluated in this article, or claim that may be made by its manufacturer, is not guaranteed or endorsed by the publisher.

## Abbreviations

DAMs: Differentially accumulated metabolites
E: Vitamin E
HPLC: High-Performance Liquid Chromatography
KEGG: Kyoto Encyclopedia Genes and Genomes
OTUs: Operational taxonomic units
Q: Quercetin
PLS-DA: Partial least squares discriminant analysis.
