# Supplementary figures and images for: Synergy of Dietary Quercetin and Vitamin E Improves Cecal Microbiota and Its Metabolite Profile in Aged Breeder Hens
Source: Front Microbiol. 2022 May 17;13:851459. doi: 10.3389/fmicb.2022.851459 (PMC9152675; doi:10.3389/fmicb.2022.851459)

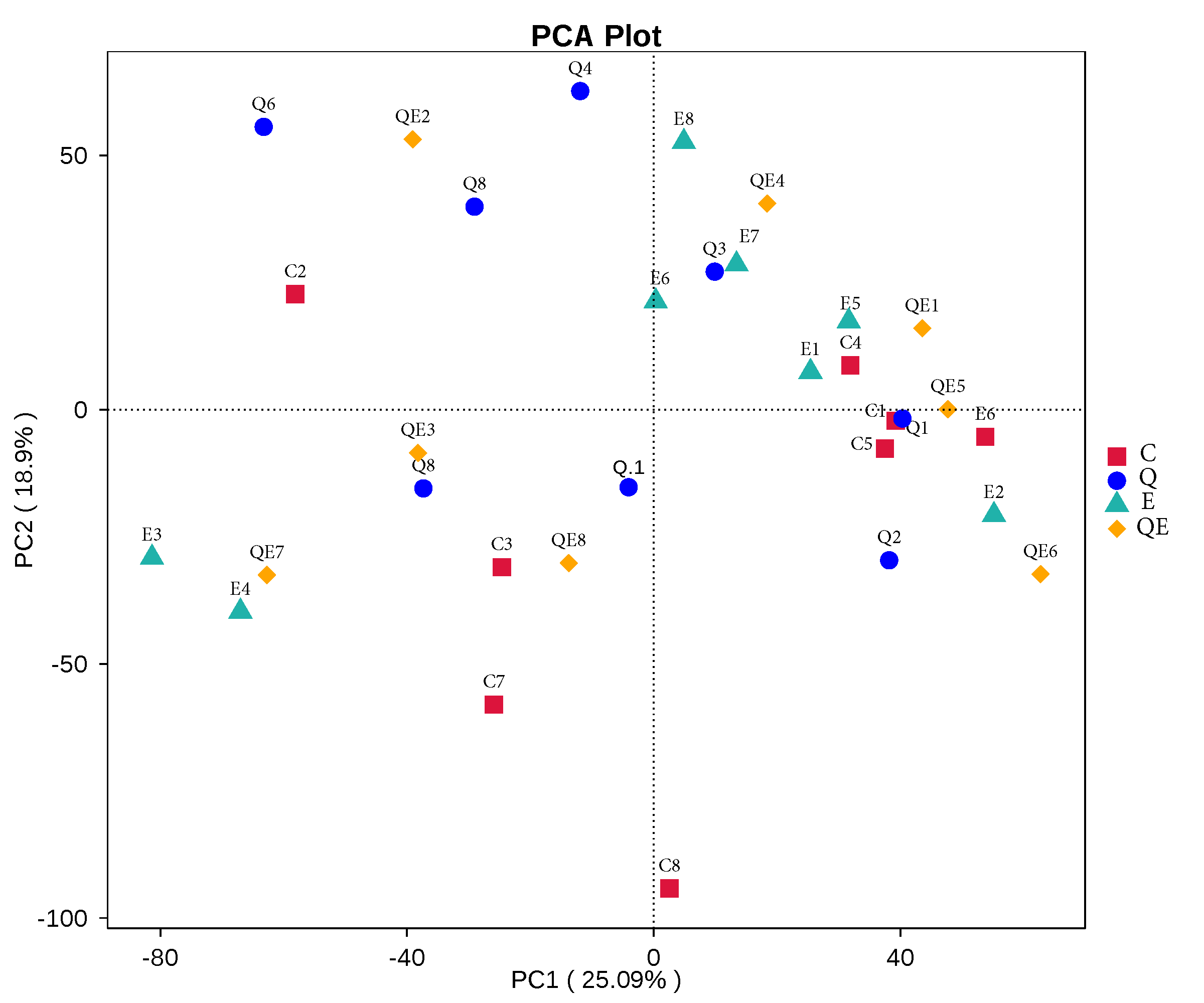

Supplement: Supplementary file 7 [file Image_1.TIFF]

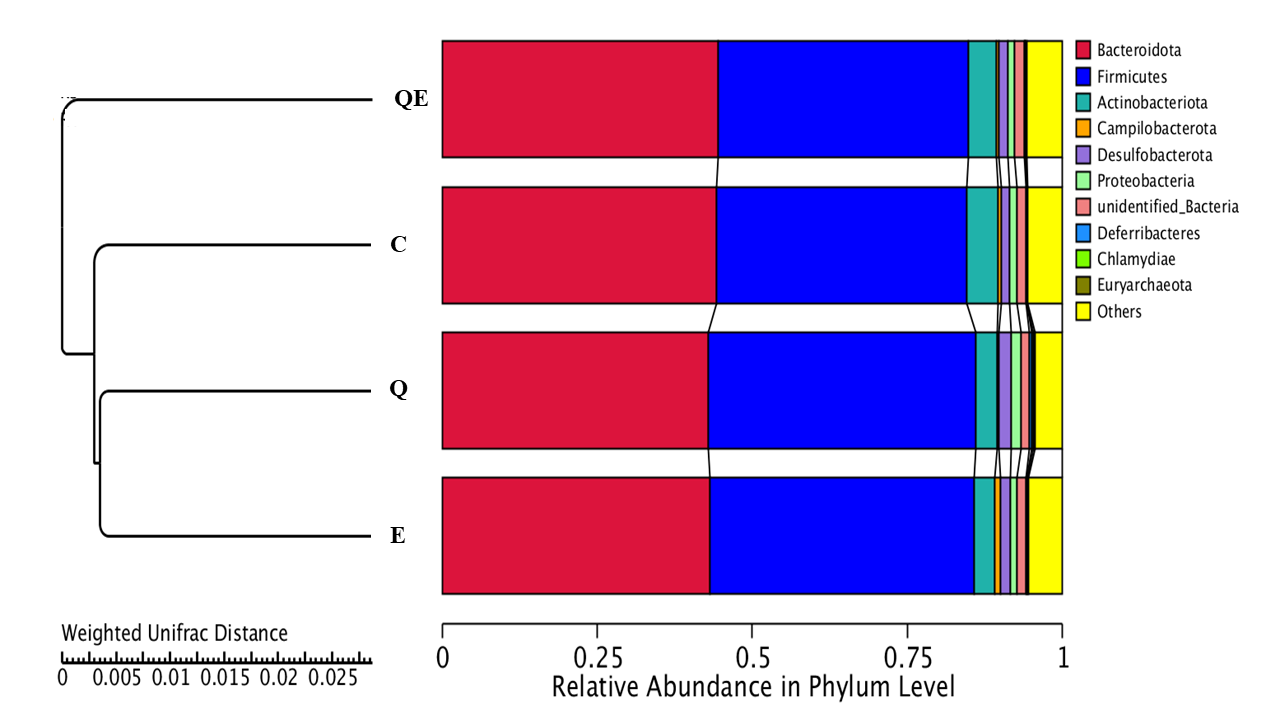

Supplement: Supplementary file 8 [file Image_2.TIF]

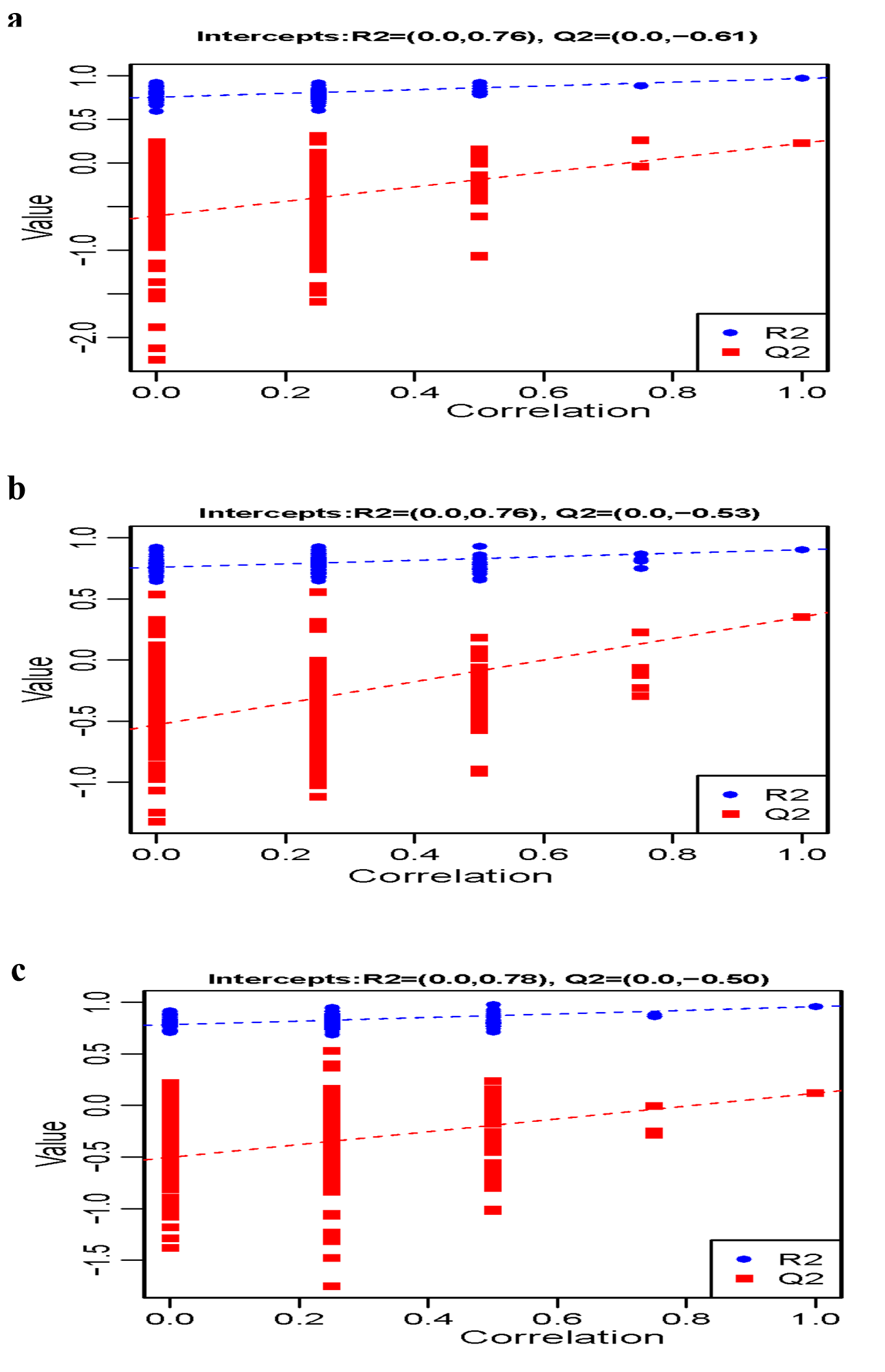

Supplement: Supplementary file 9 [file Image_3.TIF]

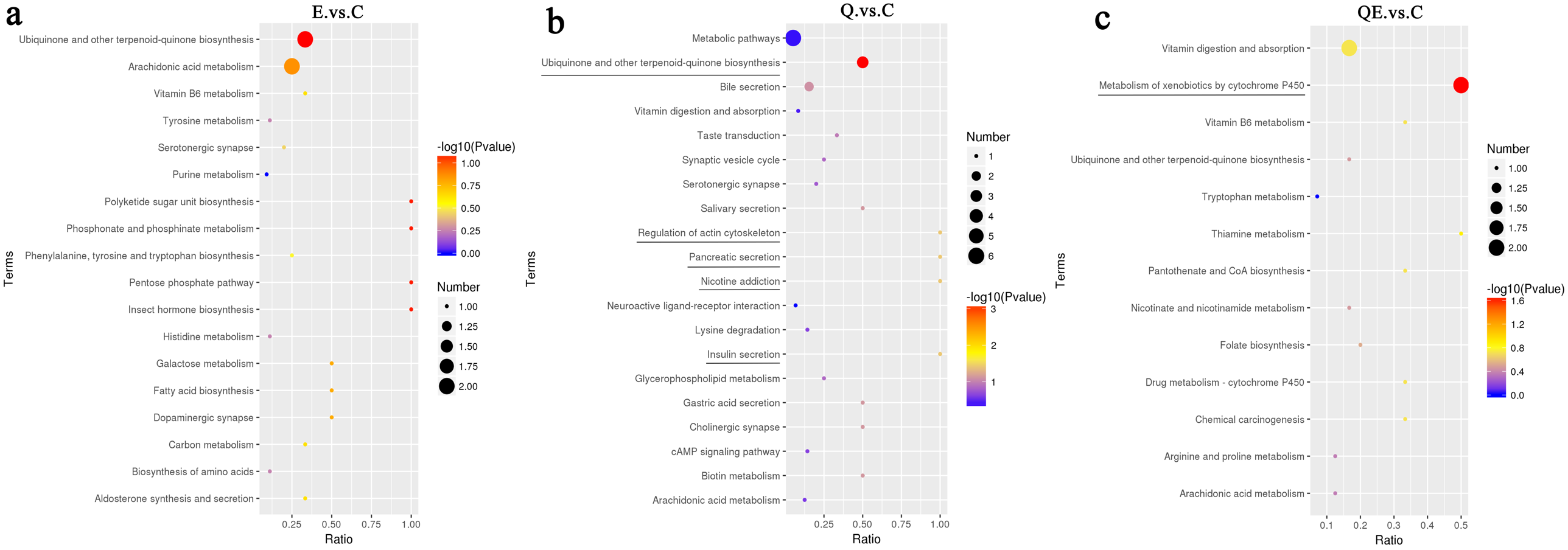

Supplement: Supplementary file 10 [file Image_4.TIF]
